# Multifunctional Polypeptide EQCN Sensors: Probing the Cysteamine-Glutathione Film Permeability with Hg(II) Ions

**DOI:** 10.3390/s8117224

**Published:** 2008-11-14

**Authors:** Maria Hepel, Julia Dallas

**Affiliations:** 1 Department of Chemistry, State University of New York at Potsdam, Potsdam, NY 13676, USA; 2 Department of Chemistry, State University of New York at Buffalo, Buffalo, NY 14260, USA

**Keywords:** Cysteamine-glutathione film, piezosensor, cysteamine SAM, EQCN, nanogravimetry

## Abstract

Multifunctional films are the basis of biosensors and play an important role in the emerging field of nanobioelectronics. In this work, films of a tripeptide glutathione (GSH) immobilized on a self-assembled monolayer of cysteamine (CA-SAM) on a quartz crystal Au piezosensor have been synthesized and characterized using electrochemical quartz crystal nanogravimetry (EQCN) with a Hg(II) ion probe. It has been found that in contrast to previously studied Au/GSH films, the Au/CA-GSH films strongly hinder the formation of Hg^0^ with bulk properties while still allowing for relatively easy permeation by Hg(II) ions. This results in complete disappearance of the sharp Hg^0^ electrodissolution peak which is observed on bare Au and Au/GSH piezosensors. The multiple-peak anodic behavior of Au/CA and bare Au is replaced by a single high-field anodic peak of mercury reoxidation in the case of Au/CA-GSH sensors. The mass-to-charge plots indicate predominant ingress/egress of Hg(II) to/from the film. The strong hindrance of CA-SAM to bulk-Hg^0^ formation is attributed to film-stabilizing formation of surface (CA)_2_Hg^2+^ complexes with conformation evaluated by *ab initio* quantum mechanical calculations of electronic structure using Hartree-Fock methods. The associates CA-GSH provide an additional functionality of the side sulfhydryl group which is free for interactions, *e.g.* with heavy metals. It is proposed that in the film, the CA-GSH molecules can assume open (extended) conformation or bent hydrogen-bonded conformation with up to four possible internal hydrogen bonds.

## 1. Introduction

Thin polypeptide films self-assembled on a Au electrode surface have been the subject of extensive studies [1-5] for the development of biosensors, nanoimprints, and for molecular electronic devices, contributing to the emerging field of nanobioelectronics [6-11]. Particularly attractive is the propensity of these films to form multifunctional active sensory materials and to interact with biocompounds. The multifunctional sensory films in future biosensors that in the perspective of developing nanofabrication technologies can be applied by nanolithography [11] to form nanosensor arrays, will play an important role in monitoring human health and diagnosing diseases. Hence, studies of chemical and physical properties of these films are of immense interest to researchers and sensor developers.

The permeability of small-polypeptide self-assembled monolayers (SAM) forming ion-channels, such as the glutathione-SAM (GSH-SAM), has been investigated using Au piezoelectrodes [1-4]. The chemically gated ion-channels have been examined using redox ion probe (hexacyanoferrate(III) ions) [1, 5], metal adatom probe [2, 4] and metal ion discharge and nucleation analysis [2]. The interactions of adsorbed GSH with Cu^2+^ have been studied [2, 12] and successful attempts to bind GSH covalently to a mercaptopropionic acid SAM on Au for heavy metal ion (Cd^2+^) sensing applications have been reported [13]. The functionalization of GSH-SAM with irreversibly bound serotonin or phenothiazine molecules (CPZ) have been observed [1]. The modification of polypeptide SAMs is desirable to control the film permeability and responsiveness to various analyte species. The use of ω-functionalized alkanethiol SAM's for the attachment of sensory molecules [14, 15] can be utilized to immobilize small polypeptides and proteins [16-18].

Studies of GSH interactions with toxic heavy metals (Hg, Cd, Pb) are important to gain better understanding of the environmental effects on human health. In particular, the elucidation of mechanisms leading to increased susceptibility to autism [19, 20], diabetes [21], and other diseases [21-26] due to diminished active GSH levels in cells and body fluids and the reduced antioxidation capacity [27] to protecting against radicals, should enable us to devise preventive measures to effectively diminish the spread and decrease the occurrence of these diseases. The involvement of GSH in counteracting heavy metal poisoning and organic peroxide neutralization is associated with its rich functionality and its key role in the GSH/GSSG system of redox regulation in living organisms.

In this work, we have investigated the immobilization of GSH on a cysteamine-SAM (CA-SAM) formed on Au piezoelectrodes by adsorptive dissociation of a disulphide, cystamine. The change in film permeability was investigated using Hg(II) ion probe which offers rich reactivity for voltammetric analysis and large molar mass easily detectable by the electrochemical quartz crystal nanogravimetry (EQCN) [28, 29]. The electrochemistry of Hg(II) on noble metal electrodes has been studied extensively on polycrystalline Au [30-37], Au nanoparticles on glassy carbon [38] and on single crystal Au(111) surface [39-49]. Hg deposition on graphite [50, 51] and platinum [52] electrodes have been investigated using EQCN. Thin Hg films on glassy carbon [53-56] and Ag microdisk [57] have been developed for heavy metal speciation by stripping voltammetric methods. In our recent studies of GSH-SAM on Au [3, 4], we have observed easy penetration of Hg(II) ions and their discharge at the bottom of ion-channels. In the present work, the films were modified by immobilization of GSH on CA-SAM to control the film permeability and to uncover the thiol group of GSH to enhance functionality of the film.

## 2. Results and Discussion

### 2.1. EQCN voltammetric analysis of Hg(II) processes at Au/CA and Au/CA-GSH piezoelectrodes

The electrochemical processes on a cysteamine-SAM (CA-SAM) modified Au electrode are hindered by blocking the access of electroactive species to the electrode surface. However, due to the short length of the carbon chain, the CA-SAM films are less compact and more disordered than aminothiol films with longer alkyl chains. On the other hand, the glutathione-SAM films (GSH-SAM) with longer peptide branches form gated ion channels with high ion permeability [1-5, 65-67]. In the following experiments, we have examined the effect of GSH bound to the CA-SAM on Hg(II) permeability and reactivity and found rather unusual behavior of these films.

On the basis of measurements performed in this work and results of our previous studies [3,4], the electrochemical reactions of a mercury probe are assigned as follows (C for cathodic and A for anodic processes):

(C_1_/A_1_) $${{\text{Hg}}^{2+}}_{\left(\text{aq}\right)}+\text{ze}={{\text{Hg}}_{\left(\text{upd}\right)}}^{+2-\text{z}}$$

(C_2_/A_2_) $${{2\text{Hg}}^{2+}}_{\text{aq}}+2{\text{e}}^{-}={\left({{\text{Hg}}_{2}}^{2+}\right)}_{\text{aq}}$$

(C_3_/A_3_) $${{\text{Hg}}^{2+}}_{\left(\text{aq}\right)}+2\text{e}={\text{Hg}}^{0}$$

(C_3_′/A_3_′) $${\left({{\text{Hg}}_{2}}^{2+}\right)}_{\text{aq}}+2\text{e}=2{\text{Hg}}^{0}$$

where the subscript upd stands for the underpotential deposit. The standard potentials for these reactions are as follows: (1) 1,163 V (NHE) [4,41-43,47], (2) 0.920 V (NHE) [4], (3) 0.854 V (NHE) [4], (3′) 0.788 V (NHE) [47]. Once a Hg^0^ film with bulk mercury properties is formed, an extensive diffusion of Hg^0^ atoms into the Au substrate is observed [3,4,38,48]. The overall process is:

(C_4_/A_4_) $${{\text{Hg}}^{2+}}_{\left(\text{aq}\right)}+n\text{Au}+2\text{e}={\text{Hg}(\text{Au})}_{\text{n}}$$

where *n* is 2, 3, or nonstoichiometric.

**Figure 1.**
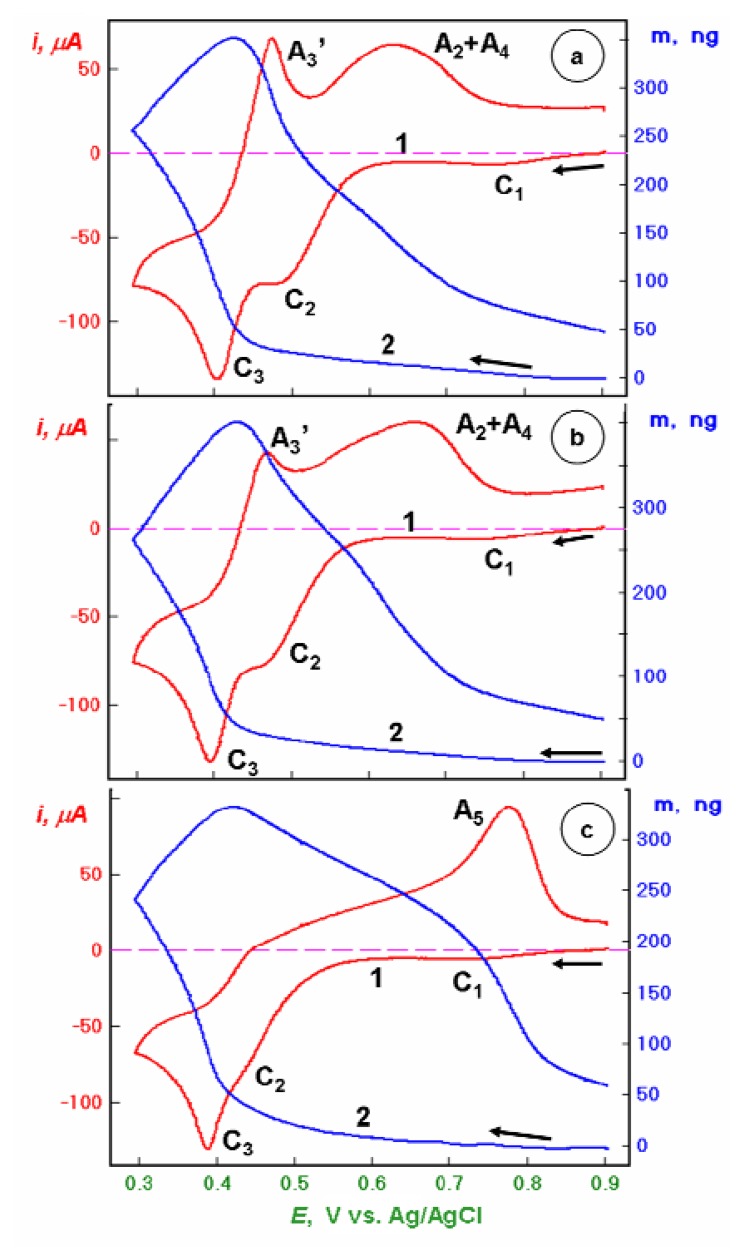
Linear potential scan voltammetric (1) and nanogravimetric (2) characteristics of piezosensors: (a) bare Au, (b) Au/CA, and (c) Au/CA-GSH, recorded in 0.1 M NaClO_4_ + 1 mM HClO_4_ + 1.5 mM Hg(ClO_4_)_2_ solution, in the potential range from *E* = +0.9 to +0.3 V vs. Ag/AgCl, at a scan rate *v* = 50 mV/s.

In Figure 1, the EQCN cyclovoltammetric characteristics for a Au, Au/CA and Au/CA-GSH piezosensors in 0.1 M NaClO_4_ + 0.001 M HClO_4_ + 1.5 mM Hg(II) are presented for the potential window from E = 0.9 to 0.2 V. It is seen that the CA-SAM decreases slightly the redox process A_2_ rate but does not hinder the Hg^0^ formation process A_3_. Interestingly, during the reverse anodic potential scan, the process A_3_′ of Hg^0^ oxidation to Hg_2_^2+^ (cf. [3]) is considerably hindered. The second anodic peak (A_2_ + A_4_) is slightly shifted toward more positive potentials.

The remarkable and unexpected change in voltammetric characteristic upon binding GSH molecules to the Au/CA film is shown in Figure 1c. Here, the redox process C_2_ is strongly hindered and the Hg^0^ reoxidation A_3_′ and processes A_2_ + A_4_ are virtually absent. Amazingly, the reduction Hg(II) → Hg^0^ remains seemingly undistorted. Almost the entire anodic reoxidation of Hg^0^ is concentrated in a new high potential process A_5_.

**Figure 2.**
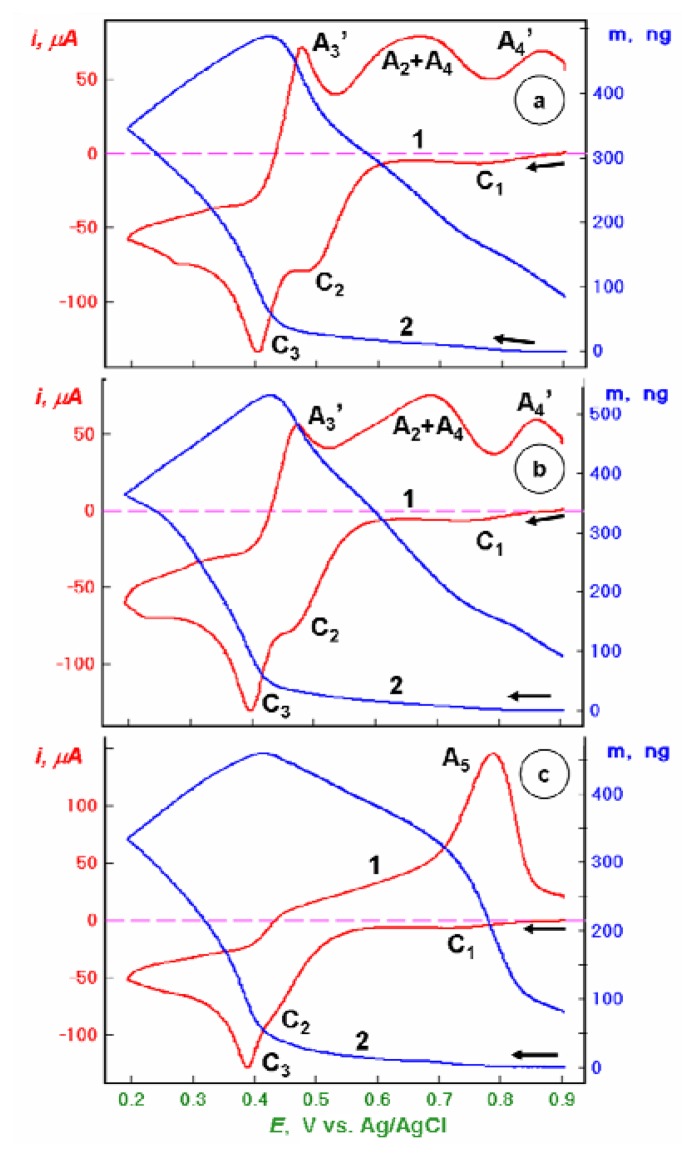
Linear potential scan voltammetric (1) and nanogravimetric (2) characteristics of piezosensors: (a) bare Au, (b) Au/CA, and (c) Au/CA-GSH, recorded in 0.1 M NaClO_4_ + 1 mM HClO_4_ + 1.5 mM Hg(ClO_4_)_2_ solution, in the potential range from *E* = +0.9 to +0.2 V vs. Ag/AgCl, at a scan rate *v* = 50 mV/s.

By expanding the potential window to E = 0.2 V (Figure 2), well below the Hg^0^ formation potential (C_3_), one observes on a bare Au electrode (Figure 2a) an extensive amalgam formation and its electrodissolution manifested by the appearance of the new anodic peak A_4_′. This peak is observed for bare Au piezosensor as well as for the Au/CA piezosensor. The mass change characteristics indicates that there is a release of mercury in the potential areas of all three anodic peaks. In the case of the Au/CA-GSH sensor, there are no new voltammetric features observed and only the increase of the anodic peak A_5_ is apparent. The mass decrease in the potential area of this peak is also increased in comparison to the characteristics in Figure 1c.

**Figure 3.**
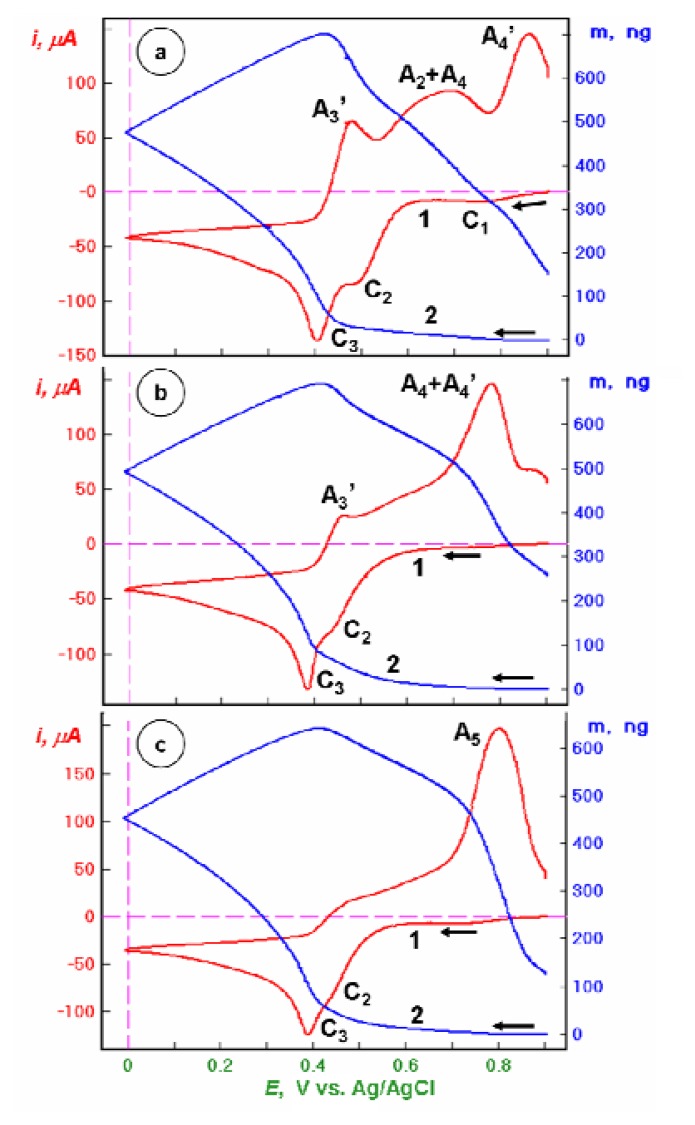
Linear potential scan voltammetric (1) and nanogravimetric (2) characteristics of piezosensors: (a) bare Au, (b) Au/CA, and (c) Au/CA-GSH, recorded in 0.1 M NaClO_4_ + 1 mM HClO_4_ + 1.5 mM Hg(ClO_4_)_2_ solution, in the potential range from *E* = +0.9 to 0 V vs. Ag/AgCl, at a scan rate *v* = 50 mV/s.

Further extension of the potential scan to E = 0 V (Figure 3) generates no new voltammetric peaks but the amount of reduced mercury increases considerably as evidenced by the increase of the second and, especially, third anodic peak in case of bare Au piezosensor and the increased charge of the combined second and third anodic peak in case of the Au/CA film. The Au/CA-GSH piezosensor shows a dominating single anodic peak with positively shifted peak potential and associated with the peak increased mass change (loss).

Note that coverages were calculated using both the charge (from current integration, with Hg_ad_ monolayer charge *Q*_mono_ = 104.9 μC per QC or 411.1 μC/cm^2^) and the apparent mass change (from frequency shift measurements). In this way, with the EQCN one can always evaluate the extent of mercury reactivity and the stream of Hg ions reaching the substrate. While the maximum surface coverage by Hg_ad_ is 0.3 for AuSG films [4], it is only a small fraction of a monolayer for AuCA or AuCA-GSH films. The thiol films form full monolayer coverage with maximum packing density of 1.6 nmol/cm^2^, for CA, which corresponds to one CA molecule per 1.5 equivalent Au surface atoms (assuming Au(111) surface with density: 2.305 nmol/cm^2^). The full monolayer coverage by the adsorbed Hg atoms corresponds the mass change *m*_mono_ = 109.1 ng (427.4 ng/cm^2^). This amount of mass is not reached durig the cathodic going scan until the cathodic current peak C3 which is due to the bulk Hg^0^ formation. This makes the mass monitoring consistent with the known voltammetric behavior.

The experimental data presented in this paper, and particularly the minute mass changes observed during the initial part of the cathodic going potential scanning (up to the voltammetric peak A_3_), do not indicate on any extensive engagement of the gold (i.e. the amalgam formation) at that stage.

In summary, we observe a single mercury electrooxidation peak for a Au/CA-GSH piezosensor and multiple peak behavior for bare Au and Au-CA piezosensors. This behavior seems to be due to the inability of Hg adatoms to form a bulk Hg^0^ phase in Au/CA-GSH films and extensive interactions of mercury species with various groups of this multifunctional film.

### 2.2. Mass-to-charge ratio

**Figure 4.**
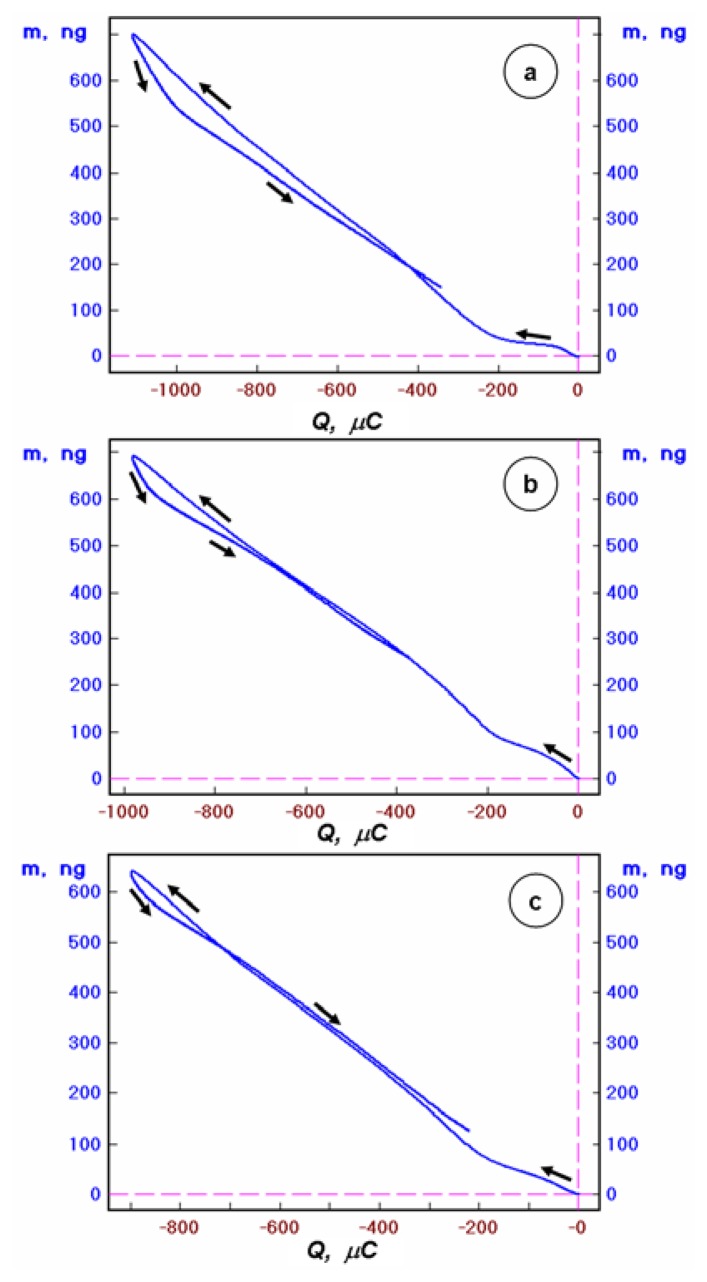
Linear potential scan EQCN plots of mass change vs. charge for: (a) bare Au, (b) Au/CA, and (c) Au/CA-GSH, recorded in 0.1 M NaClO_4_ + 1 mM HClO_4_ + 1.5 mM Hg(ClO_4_)_2_ solution, in the potential range from *E* = +0.9 to 0 V vs. Ag/AgCl, at a scan rate *v* = 50 mV/s.

The analysis of the mass-to-charge ratio indicates on the ingress/egress of Hg as the major species being exchanged at the film-solution interface. This is illustrated in Figure 4 for Au, Au/CA, and Au/Ca-GSH piezosensors. Note that the theoretical value of the derivative *∂m*/*∂Q* is: *∂m*/*∂Q* = 1.039 ng/μC for a 2-electron process (and *∂m*/*∂Q* = 2.079 ng/μC for a 1-electron process). Slightly lower experimental value of the slope *∂m*/*∂Q* is due to the concomitant exchange of electrolyte ions to fulfill the Donnan equilibrium conditions at the film-solution interface. This means that the observed slope reflects the balance of mass fluxes of cation ingress and anion egress to/from the film. The major part of cation ingress is attributed to the mercury cations but small amounts of Na^+^ and H^+^ also participate in the cation uptake. The latter contribution reduces the slope because of smaller mass (per unit charge) of these cations. The egress of anions (causing a mass loss) also contributes to the lower experimental slope. The electrolyte ion fluxes associated with the electrode potential scanning can be regarded as the double-layer effect though the electrical double-layer is here rather complex due to the presence of the semipermeable multifunctional film.

Note that in all EQCN characteristics presented in Figures 1, 2 and 3, there is seen small mass imbalance. This imbalance is due to the relatively fast scan rate (v = 50 mV/s) which is usually sufficient for establishing interfacial equilibria but when the solid state diffusion of Hg atoms in a Au substrate is taking place, some part of Hg clearly requires longer time to leave the electrode. Slower scan experiments (not shown) reduce the mass imbalance and they can be utilized if needed. The mass imbalance is dependent also on the Au particle size. Here, we have optimized the sputtered Au films to obtain *ca*. 80 nm Au nanoparticles thus making the analysis considerably faster in comparison to micrometer sized Au particulate substrates for which the pathway for solid state diffusion of Hg atoms is substantially longer. The integration of voltammetric characteristics indicates that there is also a small imbalance of charge. It is mostly associated with the same processes that lead to the mass imbalance (small amount of charge lost to the diffusion of Hg(I) intermediates to the solution is negligible due to the narrowness of the potential windows where Hg(I) ions are formed in the process C_2_ and A_3_′).

The Au-Hg alloys (amalgams) are formed mainly at potentials lower than +0.52 V vs. Ag/AgCl [49] and they may lead to Au surface roughening and pit formation [68] after Hg stripping. However, there are indications that Hg atoms may diffuse into the Au substrate even at potentials above the potential of liquid Hg^0^ monolayer formation and form gradually an amorphous Au(Hg) amalgam [48]. The mass imbalance recorded for a Au/CA-GSH electrode for potential scanning between E = +0.9 and +0.6 V (not shown) corroborates this mechanism. The metal adatom place-exchange at the SAM, e.g.:

(5) $$\text{Au}/\text{CA}+{{\text{Hg}}^{0}}_{\left(\text{ad}\right)}\to \text{Au}-\text{Hg}/\text{CA}$$

may contribute to the mercury accumulation up to the amount of CA molecules in the SAM. This process known also as the metal creep under SAM has been observed many Authors [1, 69-77] for thiols with different functional endgroups including COOH, CH_2_OH, CN, or COOCH_3_.

### 2.3. Electronic structure of CA and CA-GSH SAM films on Au

**Figure 5.**
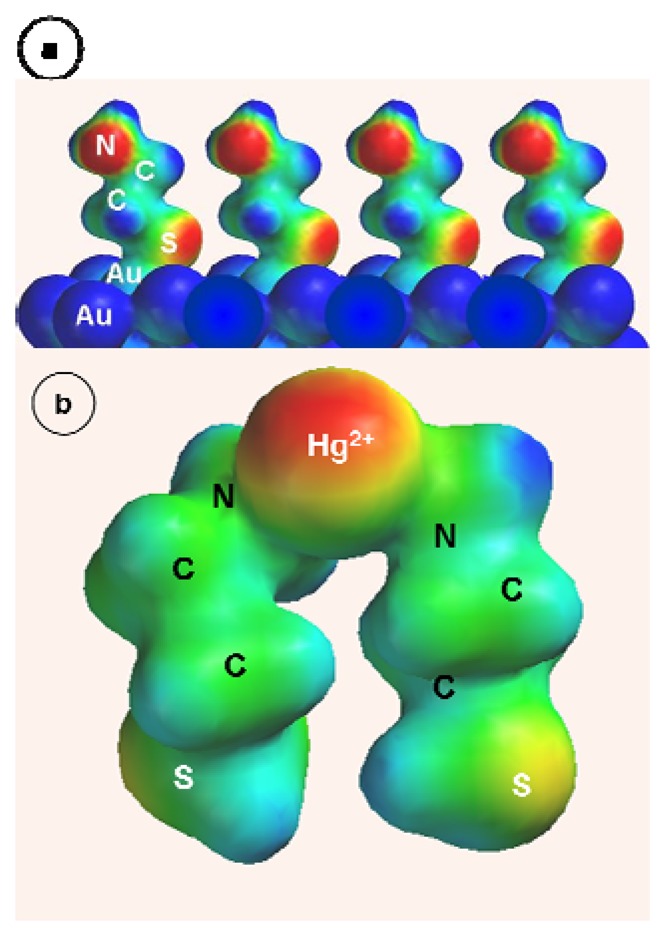
Molecular structure of a CA-SAM film on a Au piezoelectrode (a) and a Hg(CA)_2_ surface complex (b) which can form in the course of Hg^2+^ accumulation; structures calculated using HF method with 6-31G^*^ basis set and pseudopotentials for Hg and Au atoms.

The electronic structure of CA-SAM on Au has been calculated using Hartree-Fock method with 6-31G* basis set and pseudopotential for Au. The Au substrate was represented by the Au_8_-clusters per one CA molecule to reduce the calculation time. The projected film structure is depicted in Figure 5a with four such Au_8_CA structures constituting the film (part of the bottom Au atoms is not seen). The actual film structure is likely to be more disordered as no attempts have been made to account for the interplay of the solvation effects and intermolecular van der Waals forces. The CA molecules are chemisorbed on Au through the sulfur atom and with amino groups extended toward solution.

The unusual effect of strong hindrance of CA-SAM toward the bulk-Hg^0^ formation requires further elucidation. Due to the shortness of carbon backbone in cysteamine molecule which comprises only two carbon atoms, the CA-SAM is considerably more disordered than thiolate SAM's with longer alkyl chain molecules. The inherently low film order in CA-SAM's (*cf.* disorder in mercaptopropionic acid SAM [78]), in addition to the high surface mobility of adsorbed CA molecules, should allow for the nucleation of a bulk Hg^0^ phase. We have recently observed the formation of such a phase on a Au/GSH piezosensor where GSH molecules are much longer than CA molecules but have the propensity to form ion channels. It seems that there should be some stabilizing force operating in the CA-SAM's which acts as to enhance the film order and prevent the bulk-Hg^0^ formation. One such force could plausibly arise from the interaction of the CA molecules with Hg^2+^ ions. The coordination of Hg^2+^ to two amino groups of two neighboring CA molecules should reduce the lateral mobility of the adsorbed CA and increase the film order and rigidity. This structure can be further stabilized with anions. We have tested the viability of this model by applying quantum mechanical calculations of the electronic structure of various CA-Hg(II) complexes.

In Figure 5b, the electronic structure of the complex (CA)_2_Hg^2+^ is presented. The conformation of the complex is consistent with the involvement of two CA molecules adsorbed on Au in near-vertical orientation and with amino groups extended toward solution. This orientation is similar to the one observed by Raman scattering in mercaptoethanesulfonate SAM [78] where the trans conformation prevails over the gauche conformation. Note that the increased film ordering and stabilization in the latter SAM's have also been attributed to the interactions with cations [78]. The calculated N-Hg bond length is 0.211 nm and the angle N-Hg-N is 98.6^0^. The distance between two neighbor CA ligand molecules is reflected in the S-S distance which is 0.404 nm which exceeds only slightly the van der Waals distance between S atoms which is 0.363 nm. Thus, effective binding by coordination to Hg^2+^ cations provides framework for enhanced Au/CA film stability and increased film ordering. The reduction of Hg(II) would then comprise discharge followed by direct dissolution of Hg^0^ into the Au substrate (to form Au_n_Hg). In addition, the Hg^0^ adatoms may place-exchange with Au atom of the structure Au/CA leading to preferable configuration: Au-Hg/CA (*cf.* [4]). These two processes dominate over the nucleation of a bulk-Hg^0^ phase which is evident from the strong decrease of the anodic peak A′_3_ for Au/CA electrodes. It is highly unlikely that Hg^0^ islands could form on top of the CA-SAM in the process described by Kolb *et al.* [79] for dithiodipyridine SAM.

The associates CA-GSH provide an extended molecular length and the additional functionality of the side sulfhydryl group which is free for interactions, *e.g.* with heavy metals in customized sensors. We have evaluated the possible conformations of CA-GSH molecules and their electronic structure using molecular mechanics and Hartree-Fock quantum mechanical calculations.

**Figure 6.**
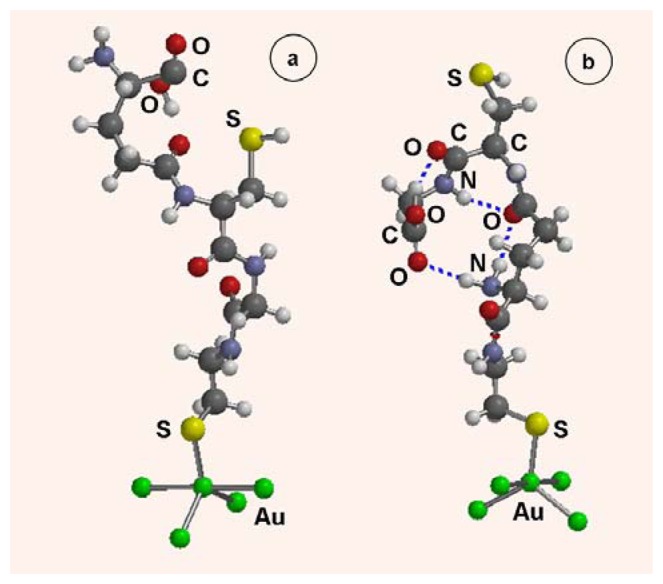
Molecular structure of a CA-GSH molecule chemisorbed on a Au cluster in an open conformation (a) and hydrogen-bonded conformation (b); hydrogen bonds are marked with dashed lines.

The CA-GSH entities were bonded to a Au_5_ metal cluster through the CA sulfur atom thus forming Au_5_CA-GSH structures. It appears that in the film, the CA-GSH molecules can assume an open (extended) conformation (depicted in Figure 6a) or a bent hydrogen-bonded conformation (depicted in Figure 6b). In the latter configuration, the SH group is extended toward the solution and up to four internal hydrogen bonds hold the molecule in the compact form. The more robust is the open conformation of Figure 6a.

## 3. Experimental Section

*Chemicals*. All chemicals used for investigations were of analytical grade purity. Cystamine NH_2_CH_2_CH_2_SSCH_2_CH_2_NH_2_ (2,2′-dithiobis(ethylamine)) and L-glutathione (GSH) were purchased from Sigma Aldrich Chemical Company and used as received. Solutions were prepared using Milli-Pore Milli-Q deionized water (conductivity *σ*= 55 nS/cm). They were deoxygenated by bubbling with purified nitrogen.

*Apparatus*. A standard electrochemical setup was employed for voltammetric measurements. It consisted of a Potentiostat/Galvanostat, Model PS-205B (Elchema, Potsdam, NY), an Electrochemical Quartz Crystal Nanobalance, Model EQCN-930, and a Data Logger and Control System, Model DAQ-716v, operating under Voltscan 5.0 data acquisition and processing software. In combined nanogravimetric and voltammetric measurements, mirror polished quartz crystal piezoresonators (QC-10Au-PB) with 5 mm diameter Au disk working electrodes, vacuum-sputtered over a 10 nm Ti underlayer, were used. The resonant frequency of these Au-piezoelectrodes was 9.975 MHz and their geometrical surface area was 0.1963 cm^2^, with the roughness factor *R* = 1.3. A standard EQCN cell with 30 mL capacity, having a side opening for sealing a quartz crystal resonator wafer (with siloxane adhesive), was used in experiments. The working electrode was polarized using a Pt wire counter electrode and its potential measured *vs.* a double-junction saturated (KCl) Ag/AgCl reference electrode. The interfacial mass changes were determined from the changes in oscillation frequency of the EQCN according to the Sauerbrey relationship [29, 58]:

(6) $$\Delta f=-\frac{2\Delta mnf_{0}^{2}}{A\sqrt{\mu_{q}d_{q}}}$$

fulfilled for thin rigid films. The latter condition was confirmed using quartz crystal immitance spectroscopy. In th above formula, the change in the resonant oscillation frequency (Δ*f*) is related to the change in the interfacial mass (*Δm*), the piezoelectrically active area (*A*), the fundamental frequency (*f*_0_), which depends on the quartz properties and resonator thickness (here: 0.166 mm), the overtone number (*n*), and to the physical properties of quartz: its density (*d_q_* = 2.648 g cm^-3^) and shear modulus (*μ_q_* = 2.947×10^11^ g cm^-1^ s^-2^). Hence, the film mass changes Δ*m* are directly related to the observed fundamental frequency shift:

(7) Δm=-0.8673Δf

provided that the solution density and viscosity remain constant during the experiment [29]. For the sake of simplicity, we will use symbol *m* to denote apparent mass changes derived from equations (1ab).

*Procedures*. The gold working electrodes sputter-deposited on a Ti-adhesion film on a quartz crystal wafer piezoresonator were cleaned with acetone, ethanol, and Milli-Q water, then etched in piranha solution (conc. H_2_SO_4_:H_2_O_2_ = 4:1) for 120 s, rinsed with distilled water and immediately immersed in 0.1 M NaClO_4_ + 1 mM HClO_4_ solution. The Au/CA modified electrodes were prepared by immersion of freshly prepared metal electrodes in 10 mM cystamine + 0.1 M HClO_4_ solution for 20 minutes which resulted in dissociative adsorption of the disulphide and formation of reproducible films. The Au/CA-GSH films were synthesized by incubating the Au electrode in 10 mM CA solution in 75% ethanol for 20 min., followed by rinsing with absolute ethanol and immersing in 10 mM GSH solution for 30 min. The electrodes were rinsed with 0.1 M NaClO_4_ + 1 mM HClO_4_ solution and immediately used for testing. The fast and effective CA-GSH interactions are attributed to the electrostatic and hydrogen bonding between the amine and carboxylate functional groups. Note that extensive hydrogen bonding has been recently discovered in thiolate-linked Au-nanoparticle networks by Zhong et al. [59-62]. The electrodes were emersed from the analyte solutions under a protecting potential (*E*_cond_ = +0.9 V vs. Ag/AgCl) to avoid any uncontrolled mercury deposition and/or amalgam formation. The experiments were performed at room temperature, 22°C.

Quantum mechanical calculations of electronic structure for adsorbed CA, CA-GSH and CA-Hg(II) complexes were performed using modified Hartree-Fock methods [63] with 6-31G* basis set and pseudopotentials, semi-empirical PM3 method, and density functional theory (DFT) with B3LYP functional and 6-31G* basis set, embedded in Wavefunction Spartan 6 [63, 64]. The electron density and local density of states (LDOS) are expressed in atomic units, au^3^, where 1 au = 0.529157 Å and 1 au^3^ = 6.749108 Å^-3^.

## 4. Conclusions

The cysteamine SAM's on Au obtained by dissociative chemisorption of a disulphide, cystamine, provide sufficiently dense films that can be utilized for assembling multilayer and multifunctional sensory films. The Au/CA and Au/CA-GSH piezosensors have been synthesized and the permeability tests performed by the EQCN method indicate that the films are permeable to Hg(II) ion probe but successfully prevent the formation of a bulk-Hg^0^ phase. It is proposed that this is due to the film-ordering and stabilizing formation of surface (CA)_2_Hg^2+^ complexes with electronic structure and conformation evaluated by *ab initio* quantum mechanical calculations. Even stronger film-stabilizing properties provide GSH moieties in the Au/CA-GSH associates. In this situation, the anodic discharge of reduced mercury which normally presents a multi-peak structure (e.g. for bare Au or Au/GSH films), has been found to generate only one high-field peak for Au/CA-GSH piezosensors. Since the mass change associated with this peak is larger than the equivalent monolayer mass of Hg^0^, the anodic process must comprise electroxidation of Hg atoms from both the Au-Hg/CA-GSH associates and the underlying amalgam. The functional groups introduced to the film with tripeptide glutathione, including its free sulfhydryl group, amine, and carboxylate ligands, can be utilized in designing sensors, e.g. for heavy metals or biocompounds interacting with glutathione.
